# Bio-organic amendment enhances apple yield and nutritional quality in degraded sandy soils

**DOI:** 10.3389/fpls.2026.1834274

**Published:** 2026-05-21

**Authors:** Mingjing Li, Chunguang Bao, Rui Luo, Guorui Li, Jianjun Di, Cheng Wang, Yong Zhao, Fenglan Huang

**Affiliations:** 1 College of Life Science and Food, Inner Mongolia Minzu University, Tongliao, China; 2 Jilin Provincial Key Laboratory of Tree and Grass Genetics and Breeding, College of Forestry and Grassland Science, Jilin Agricultural University, Changchun, China; 3 Tongliao Agricultural and Animal Product Quality Safety Center, Inner Mongolia, Tongliao, China; 4 College of Life Science, Baicheng Normal University, Baicheng, China; 5 Jilin Provincial Key Laboratory of Western Jilin’s Clean Energy, Baicheng Normal University, Baicheng, China; 6 Key Laboratory of Castor Breeding of State Ethnic Affairs Commission, Tongliao, China; 7 Inner Mongolia Key Laboratory of Castor Breeding and Comprehensive Utilization, Tongliao, China; 8 Inner Mongolia Engineering Research Center of Industrial technology Innovation of Castor, Tongliao, China; 9 Inner Mongolia Industrial Engineering Research Center of Universities for Castor, Tongliao, China

**Keywords:** castor bean enzyme biofertilizer, malus pumila, quality, soil enzyme activity, soil fertility, yield

## Abstract

**Introduction:**

‘Saiwaihong’ apple (*Malus pumila ‘Saiwaihong’*) is a premium cultivar cultivated in the Horqin sandy land of Inner Mongolia, where soils are characterized by low organic matter content and poor nutrient retention capacity. Castor bean enzyme biofertilizer (CB), produced through microbial fermentation of castor meal, has shown promise in enhancing crop productivity, but its effects on fruit tree performance and underlying mechanisms remain inadequately characterized.

**Methods:**

A two-year field experiment was conducted using 7-year-old ‘Saiwaihong’ apple trees in a randomized complete block design with six treatments: unfertilized control (CK), cattle manure (N), compound fertilizer (F), and three CB application rates. Soil physicochemical properties, enzyme activities, fruit yield components, and quality parameters were systematically evaluated.

**Results:**

In this study showed that CB application uniquely reduced soil pH from strongly alkaline toward near-neutral conditions and elevated soil organic matter by 50.9-70.6% above the control. Available phosphorus increased dramatically under B1 and B2, accompanied by sustained enhancement of hydrolytic enzyme activities and suppression of polyphenol oxidase. The B2 treatment achieved superior yield enhancement through increased individual fruit size. Most notably, CB treatments elevated fruit vitamin C content by 101.4-135.9% across both years-an effect size substantially exceeding reported values for conventional amendments. Strong positive correlations between soil enzyme activities and fruit quality parameters indicated functional coupling between enhanced soil biological functioning and improved nutritional quality. Our findings demonstrate that moderate CB application (21, 500 kg·ha-1) optimally enhanced soil properties, enzymatic activity, yield, and fruit quality in ‘Saiwaihong’ apple production. These benefits are mediated through improved soil physicochemical conditions and enhanced biological nutrient cycling.

**Discussion:**

These findings position CB as a promising strategy for building longterm soil health and supporting sustainable orchard management in nutrientpoor sandy soils, offering theoretical guidance for green agriculture development.

## 1 Introduction

*Malus pumila* ‘Saiwaihong’ (also known as ‘Jinxiu Haitang’ or ‘Jixin Fruit’) is a renowned premium characteristic agricultural product of Tongliao City, Inner Mongolia. Renowned for its aromatic, crisp, and sweet flavor profile, this cultivar has gained substantial popularity among consumers (Li et al., 2021; Tian et al., 2025). In recent years, the cultivation area of ‘Saiwaihong’ apple has expanded considerably, accompanied by continuous increases in production. However, this growth concurrently presents potential risks pertaining to orchard management practices and market valuation, underscoring an urgent need to enhance fruit quality and market competitiveness (Li et al., 2021a). Tongliao City, situated in the eastern part of Inner Mongolia Autonomous Region, lies within the hinterland of the Horqin sandy land. The soils exhibit zonal distribution from south to north: the central region is predominantly characterized by gray meadow soils and aeolian sandy soils; the southern region by cinnamon soils and castano-cinnamon soils; and the northern region by chestnut soils and chernozems (Zhang et al., 2008; Yao et al., 2013). Consequently, substantial areas are plagued by poor nutrient retention capacity and low soil organic matter content (Xu et al., 2025). This edaphic challenge is compounded by a lack of technical knowledge among many growers regarding optimal cultivation practices for this specific cultivar, leading to extensive management with low input. Such practices result in unstable yield per plant and per unit area, as well as inconsistent fruit quality (Lynch, 2022; Hulshof and Spasojevic, 2020). Therefore, improving soil conditions, enhancing soil organic matter through the judicious application of fertilizers, and consequently elevating fruit quality represent indispensable pathways for the sustainable development of the ‘Saiwaihong’ apple industry.

Fertilization is a critical management practice for enhancing the productivity of ‘Saiwaihong’ apples. Although chemical fertilizers provide essential nutrients that significantly promote tree growth and development, their over-application can detrimentally affect fruit quality, escalate production costs, and pose risks to both soil health and the atmospheric environment (Ahmed et al., 2024a; Wei et al., 2024; Tang, 2024). Consequently, optimizing fertilizer regimes-including source, rate, and their interaction with yield and quality-is fundamental for achieving sustainable, high-quality production of this cultivar. Organic amendments also play a vital role in this cultivation system, as they can not only significantly increase yield but also improve soil conditions, enhance fertilizer use efficiency, and reduce pollution (Shu et al., 2022; Omokaro et al., 2024). For instance, Jakhro et al. (2025a) reported that agronomic practices such as crop residues, novel organic soil amendments, and diversified cover cropping systems contribute to the enhancement of key ecosystem functions in apple orchard management by elevating the soil organic carbon pool. Furthermore, Jakhro et al. (2025b) showed that soil physicochemical properties in apple orchards display clear vertical stratification, with the combined manure and chemical fertilizer treatment (MCF) being most effective at improving surface soil water retention, carbon sequestration, and nutrient availability. Consequently, organic and bio-organic fertilizers have gained attention for their potential to improve soil health, nutrient efficiency, and environmental outcomes.

Castor bean meal, a byproduct of oil extraction from *Ricinus communis* L., is rich in protein, crude fiber, and essential mineral elements. Castor bean enzyme biofertilizer (CB), produced through microbial fermentation of castor meal, possesses properties of both microbial and organic fertilizers, improving the soil habitat, solubilizing nutrients, and supporting high-quality crop production. For example, Lei et al. (2026) demonstrated that the application of CB biofertilizer to cadmium-contaminated soil alleviates cadmium toxicity in plants. Li et al. (2024) reported that application of CB biofertilizer in peanut cultivation enhanced sandy soil fertility, soil enzyme activity, peanut yield, and unsaturated fatty acid content. In addition, Li et al. (2025) further demonstrated that applying the CB biofertilizer during tartary buckwheat cultivation not only enhanced the soil physicochemical environment of the planting area but also indirectly increased the economic value of buckwheat by-products by improving crop quality. Similarly, CB has been shown to improve the yield and quality of pepper, spinach, and maize by enhancing the soil nutrient environment (Tian et al., 2022). These findings underscore that targeted soil amelioration with bio-organic fertilizers like CB is a promising strategy for achieving sustainable, high-quality agricultural output.

Castor bean enzyme biofertilizer (CB) has demonstrated potential in enhancing the yield and quality of economic crops such as peanut, tartary buckwheat, and pepper (Li M et al., 2024; Li M et al., 2025). However, its efficacy and underlying mechanisms in widely consumed fruit crops remain critically under-investigated. This lack of systematic evidence hinders the optimization and broader adoption of CB within sustainable fruit production systems. To address this gap, we conducted a two-year field trial using the ‘Saiwaihong’ apple as a model system, comparing the effects of chemical fertilizer, cattle manure, and varying application rates of CB. We systematically evaluated their impacts on soil physicochemical properties, soil enzyme activities, and the resultant apple yield and fruit quality, testing the hypothesis that the benefits of CB for apple productivity and quality are mediated through improved soil physicochemical conditions and enhanced soil enzyme activities. This study aims to elucidate the agronomic value of CB in ‘Saiwaihong’ apple production and to provide practical insights for reducing reliance on synthetic fertilizers, thereby promoting soil health-based sustainable orchard management.

## 2 Materials and methods

### 2.1 Experimental site

A two-year field experiment was conducted from June 2024 to August 2025 at the Experimental Research Center of the Tongliao Forestry and Grassland Science Institute (122°01′E, 44°04′N; altitude 181.4 m). The site experiences a temperate continental climate, with a mean annual temperature of 6.5 °C and mean annual precipitation of 395 mm, mostly occurring in July and August. The soil is classified as a sandy loam, with initial physicochemical properties as follows: pH 8.62, organic matter 17.0 g·kg^-1^, total nitrogen 1.21 g·kg^-1^, total phosphorus 0.46 g·kg^-1^, total potassium 23.2 g·kg^-1^, alkaline-hydrolyzable nitrogen 53 mg·kg^-1^, available phosphorus 6.8 mg·kg^-1^, and available potassium 163 mg·kg^-1^.

### 2.2 Experimental materials

#### 2.2.1 Plant materials and experimental site

This study utilized 7-year-old ‘Saiwaihong’ apple trees (*Malus pumila ‘Saiwaihong’*). The plant materials were provided by the Experimental Center of the Tongliao Forestry and Grassland Science Research Institute in Tongliao, Inner Mongolia, China.

#### 2.2.2 Experimental fertilizers

Cold-pressed castor bean (*Ricinus communis* L.) meal and a fermentation inoculant package were supplied by the Key Laboratory of Castor Bean Breeding, State Ethnic Affairs Commission. The castor bean enzyme biofertilizer (CB) was produced by fermenting the castor meal with the inoculant at 37 °C for 28 days. The physicochemical properties of the finished CB were as follows: pH 6.30, organic matter 814.00 g·kg^-1^, organic carbon 472.10 g·kg^-1^, total nitrogen (N) 6.06 g·kg^-1^, total phosphorus (P) 4.25 g·kg^-1^, total potassium (K) 8.20 g·kg^-1^, alkali-hydrolyzable N 894.00 mg·kg^-1^, available P 55.70 mg·kg^-1^, and available K 7187.0 mg·kg^-1^. The fermentation inoculant consisted of a consortium of seven beneficial microbial strains, including *Bacillus subtilis*, *Saccharomyces cerevisiae*, and *Spirulina platensis*, as well as eight soil enzymes such as Urease, Phosphatase, Sucrase, Catalase, Acid invertase, Polyphenol oxidase, Protease, and Cellulase. Chemical Fertilizer and Organic Amendment The water-soluble chemical fertilizer used in this experiment was a commercial product (Jiashili^®^, China) with a guaranteed analysis of total nutrients (N + P_2_O_5_ + K_2_O) ≥ 45% (12-18-15) and total sulfur (S) ≥ 10%. Well-decomposed cattle manure (N) was obtained from the Tongliao Bureau of Agriculture and Animal Husbandry.

### 2.3 Experimental design

A randomized complete block design was employed, comprising six fertilization treatments, each with five trees and three replications. The treatments included: CK (no fertilizer control), N (Organic manure), F (compound fertilizer), and B1, B2, and B3 (castor bean enzyme biofertilizer applied at three different rates). The application rate of the CB biofertilizer was determined based on the application rates of chemical fertilizer, cattle manure, and the findings of our previous studies. The total application rates of nitrogen (N), phosphorus (P), and potassium (K) for each treatment are detailed in **Table 1**. The application rates of the castor bean enzyme biofertilizer were determined based on the soil improvement and fertility enhancement techniques for sandy orchards in the Tongliao region, as described by Musei et al. (2024). The organic manure rate followed local farming practices, while the compound fertilizer rate was guided by the fertilization recommendations for ‘Saiwaihong’ apples issued by the Ministry of Agriculture and Rural Affairs, China. Critically, as shown in **Table 1**, the application rates for treatments F, N, and B2 were designed to ensure approximately equivalent total inputs of N, P_2_O_5_, and K_2_O. This design aimed to enable a meaningful comparison of the effects of different fertilizer types under comparable nutrient supply levels.

**Table 1:** Nitrogen, phosphorus, and potassium (N, P, K) contents of each treatment.

Treatment	Rate of fertilizer application (kg·ha^-1^)	N (kg·ha^-1^)	P_2_O_5_ (kg·ha^-1^)	K_2_O (kg·ha^-1^)
CK	0	0	0	0
N	12, 500	45.72	65.40	56.45
F	375	45.00	67.50	56.25
B1	10, 750	23.79	32.71	27.16
B2	21, 500	47.59	65.42	54.31
B3	43, 000	95.18	130.84	108.62

CK, Fertilizer application rate of 0 kg·ha^-1^; N, Manure application rate of 12, 500 kg·ha^-1^; F, Chemical fertilizer application rate of 375 kg·ha^-1^; B1, CB application rate of 10, 750 kg·ha^-1^; B2, CB application rate of 21, 500 kg·ha^-1^; B3, CB application rate of 43, 000 kg·ha^-1^.

**Figure 1:**
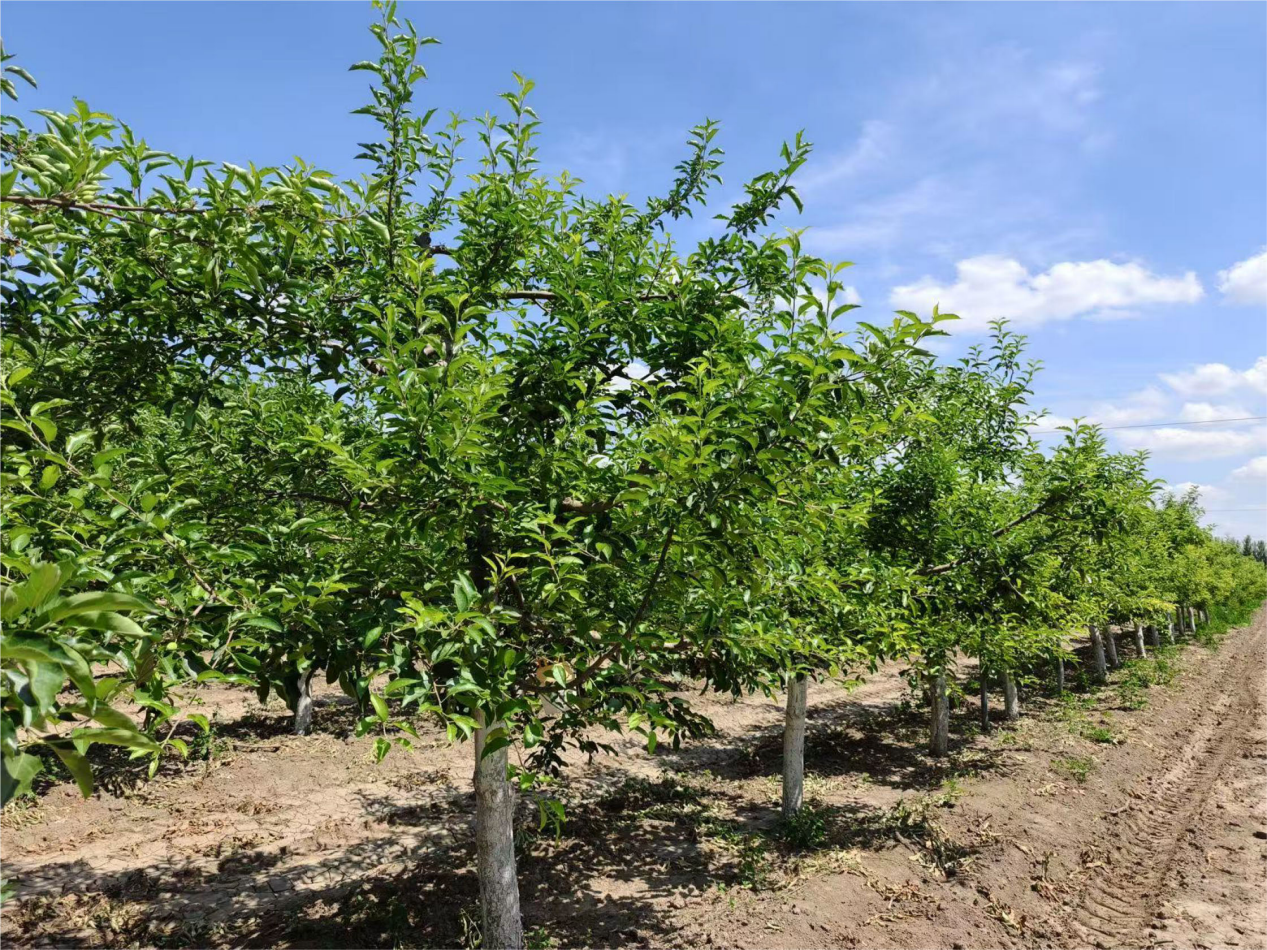
Field growth photograph of Saiwaihong apple trees.

Fertilizers were applied in two splits. The first application was conducted on June 8, 2024, and June 10, 2025 (during the young fruit stage). Fertilizer was incorporated into four evenly spaced pits dug in a circle 20 cm from the trunk. Each pit measured 20 cm × 20 cm in length and width, with a depth of 40 cm. The application rates per tree were as follows: CK received no fertilizer; N received 5 kg of organic manure; F received 0.15 kg of compound fertilizer; B1 received 4.3 kg of castor bean enzyme biofertilizer; B2 received 8.6 kg; and B3 received 12.9 kg. The fertilizer was thoroughly mixed with the soil before backfilling. The second fertilization was performed on July 6, 2024, and July 10, 2025 (during the fruit expansion stage), following the same procedure and application rates. No additional fertilizers were applied during the growing season, and all other field management practices followed local conventional protocols. Field growth photograph of Saiwaihong apple trees is shown in **Figure 1**.

### 2.4 Sample preparation

Fruits were harvested at full maturity on September 15, 2024, and September 21, 2025. All mature ‘Saiwaihong’ apples from each treatment were collected and promptly placed in preservation boxes maintained at 4 °C. Subsequently, the yield per plot, average yield per tree, mean single fruit weight was determined.

Following fruit harvest, soil samples were collected from each fertilization treatment for analysis. Surrounding each tree within a treatment, four sampling points located 20 cm from the trunk were established. After removing the surface litter and topsoil, soil samples from the 20–40 cm plow layer were collected using a shovel. Within each plot, five soil cores were randomly collected along an “S”-shaped sampling path using a soil auger and combined to form one composite sample. Visible plant debris and stones were removed, and the soil samples were subsequently passed through a 2-mm sieve. A portion of each sample was air-dried for the analysis of soil physicochemical properties, while the remaining subsample was stored at 4 °C for subsequent determination of soil enzyme activities.

### 2.5 Measurements

#### 2.5.1 Determination of soil nutrient contents

Soil physicochemical indicators were measured after fruit harvest across different fertilization treatments. Specifically, soil pH was determined using the potentiometric method. Soil organic matter content was measured using the potassium dichromate external heating method (Virtanen et al., 2017). Total nitrogen (TN) content was analyzed via the Kjeldahl method (Yang et al., 2017). Total phosphorus (TP) content was determined using the molybdenum-antimony anti-colorimetric method (Sa et al., 2025). Total potassium (TK) content was measured by flame photometry (Li J et al., 2025). Alkali-hydrolyzable nitrogen (AN) content was determined using the alkaline hydrolysis diffusion method (Chen et al., 2016). Available phosphorus (AP) content was measured using the NaHCO_3_-molybdenum-antimony anti-colorimetric method, and available potassium (AK) content was determined using the NH_4_OAC-flame photometry method (Ameer et al., 2024).

#### 2.5.2 Determination of soil enzyme activities

Soil enzyme activities were assayed using commercial detection kits (Nanjing Cavens Biotechnology Co., Ltd., China), strictly following the manufacturer’s instructions. Urease (URE) activity was determined using the sodium phenate-sodium hypochlorite colorimetric method. Sucrase (SUC) activity was measured via the 3, 5-dinitrosalicylic acid (DNS) colorimetric method. Alkaline phosphatase (ALP) activity was assessed using the disodium phenyl phosphate colorimetric method. Protease (PRO) activity was analyzed using the copper salt complexation colorimetric method. Polyphenol oxidase (PPO) activity was determined using the catechol colorimetric method. Dehydrogenase (DEH) activity was measured using the TTC (2, 3, 5-triphenyltetrazolium chloride) spectrophotometric method.

#### 2.5.3 Determination of ‘Saiwaihong’ apple yield parameters

The yield per plot (total weight of all harvested ‘Saiwaihong’ apples) was recorded, and the average yield per tree was calculated based on the plot yield and the number of trees per treatment. For each treatment, ten representative ‘Saiwaihong’ apple fruits were selected from each of the three replicates, resulting in a total of 30 apples per treatment. The individual fruit weight was measured using an electronic balance with an accuracy of 0.001 g. The mean single fruit weight was then calculated as the average of these ten fruits.

#### 2.5.4 Determination of ‘Saiwaihong’ apple fruit appearance quality parameters

For each treatment, ten representative ‘Saiwaihong’ apple fruits were selected to measure fruit vertical diameter and transverse diameter using an electronic digital caliper (Model: LED150). The fruit shape index was calculated as the ratio of vertical diameter to transverse diameter. Fruit maturity (peel color) was evaluated for each treatment according to the Regional Standard of Inner Mongolia Autonomous Region DB15/T 2942-2023.

#### 2.5.5 Determination of internal quality parameters of ‘Saiwaihong’ apples

The internal quality parameters of harvested ‘Saiwaihong’ apples from each fertilization treatment were determined as follows: Fruit moisture content was measured using the direct drying method (Kahraman et al., 2021). Vitamin C content was quantified by high-performance liquid chromatography (HPLC) (Wu et al., 2023). Total soluble solid (TSS) content was determined using a hand-held refractometer (Shanghai Jiahang Instruments Co., Ltd., China). Soluble sugar content was analyzed using the 3, 5-dinitrosalicylic acid (DNS) colorimetric method (Yang et al., 2019). Titratable acidity (TA) was measured using the acid-base titration method (Ban and Xu, 2020). Flesh firmness was determined using a fruit firmness tester (Model FT-327, Facchini, Italy).

### 2.6 Statistical analysis

Experimental data were compiled and organized using Microsoft Excel 2016, which was also employed for table generation. Bar graphs were created using GraphPad Prism version 10 (GraphPad Software, San Diego, CA, USA). Statistical analyses were performed using SPSS version 27.0 (IBM Corp., Armonk, NY, USA). Differences among fertilization treatments were assessed using one-way analysis of variance (ANOVA), followed by Tukey’s multiple comparison test at a significance level of *P <* 0.05.* A* Spearman’s rank correlation analysis was conducted, with statistical significance set at *P* < 0.05, *P* < 0.01, and *P* < 0.001. The increase rate for different fertilization treatments was calculated as (treatment value - control value)/control value × 100%, while the decrease rate was calculated as (control value - treatment value)/control value × 100%. All data are means ± standard error (n = 3).

## 3 Results

### 3.1 Effects of different fertilization treatments on soil physicochemical properties

**Figure 2:**
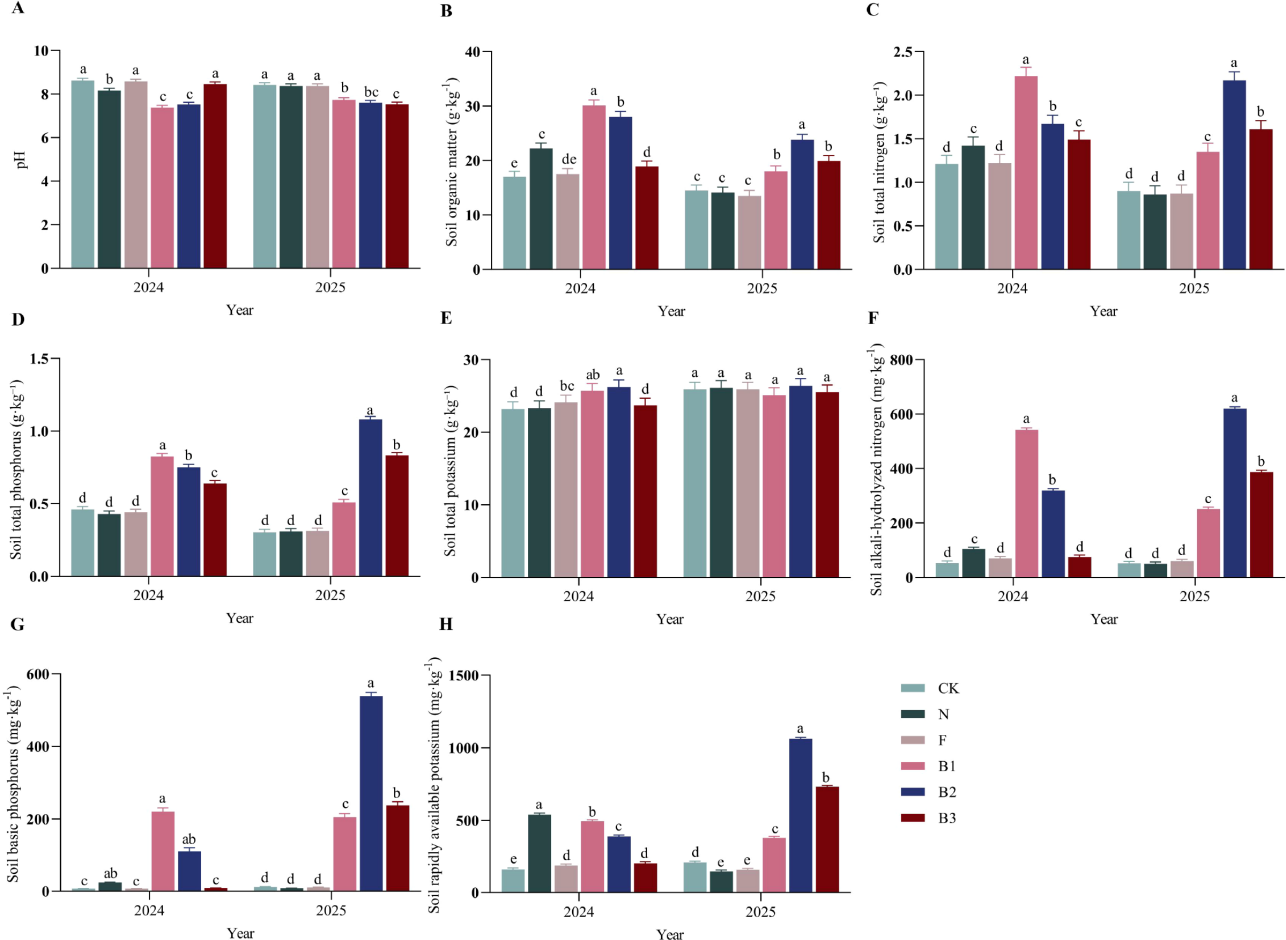
Effect of different fertilization treatments on soil nutrients content of ‘Saiwaihong’ apple in 2024 and 2025. **(A)** Soil pH. **(B)** Soil organic matter (SOM). **(C)** Total nitrogen (TN). **(D)** Total phosphorus (TP). **(E)** Total potassium (TK). **(F)** Soil alkaline-hydrolyzable nitrogen (AN). **(G)** Soil available phosphorus (AP). **(H)** Soil available potassium (AK). Data are means ± standard error (n = 3). Different lowercase letters above bars indicate significant differences among treatments within the same year (Tukey’s HSD test, *P* < 0.05). CK: Fertilizer application rate of 0 kg·ha^-1^; N: Manure application rate of 12, 500 kg·ha^-1^; F: Chemical fertilizer application rate of 375 kg·ha^-1^; B1: CB application rate of 10, 750 kg·ha^-1^; B2: CB application rate of 21, 500 kg·ha^-1^; B3: CB application rate of 43, 000 kg·ha^-1^.

Fertilization regimes distinctly shaped soil chemical properties over the two-year study (**Figure 2**). Castor bean enzyme biofertilizer (CB) application uniquely reduced soil pH and enhanced organic matter and nitrogen availability. In 2024, the CB treatments (B1, B2) significantly lowered pH by 14.4% and 12.8%, respectively, compared to the unfertilized control (CK). Although this pH reduction was less pronounced in 2025 (8.2-10.6% lower than CK), the pH in CB-treated plots remained significantly below that of all other fertilizer treatments. Soil organic matter (SOM) was consistently higher in plots receiving CB. Across both years, the B1 and B2 treatments increased SOM by an average of 70.6% and 50.9% over CK, respectively, despite a slight overall decline in SOM from 2024 to 2025. Nutrient responses were treatment-specific. Total nitrogen (TN) was significantly higher in all CB-treated plots (B1, B2, B3) compared to CK in both years, with average increases of 66.8% for B1, 89.6% for B2, and 51.0% for B3. Total phosphorus (TP) was also significantly elevated in all CB treatments, an effect that persisted into the second year. In contrast, total potassium (TK) was unaffected by any fertilization treatment throughout the study. CB treatments consistently improved nitrogen availability. In 2024, the B1 treatment resulted in the highest alkaline-hydrolyzable nitrogen (AN) content, whereas in 2025, the highest AN was observed under B2. The positive effect of CB was even more pronounced for available phosphorus (AP). Across the two years, the B1 and B2 treatments increased AP by an average of 2407.6% and 3009.9% over CK, respectively. Soil available potassium (AK) dynamics differed from those of nitrogen and phosphorus. In 2024, the cattle manure treatment exhibited the highest AK, surpassing CK by 231.3%. However, by 2025, the B2 treatment resulted in the greatest AK content (a 405.7% increase over CK), with the other CB treatments also maintaining higher AK levels than non-CB fertilized plots. Collectively, these results indicate that castor bean enzyme biofertilizer effectively modifies the soil nutrient landscape. Its beneficial effects on overall soil nutrient status, particularly on organic matter and nitrogen availability, became more pronounced in the second year of application.

### 3.2 Effects of different fertilization treatments on soil enzyme activities

**Figure 3:**
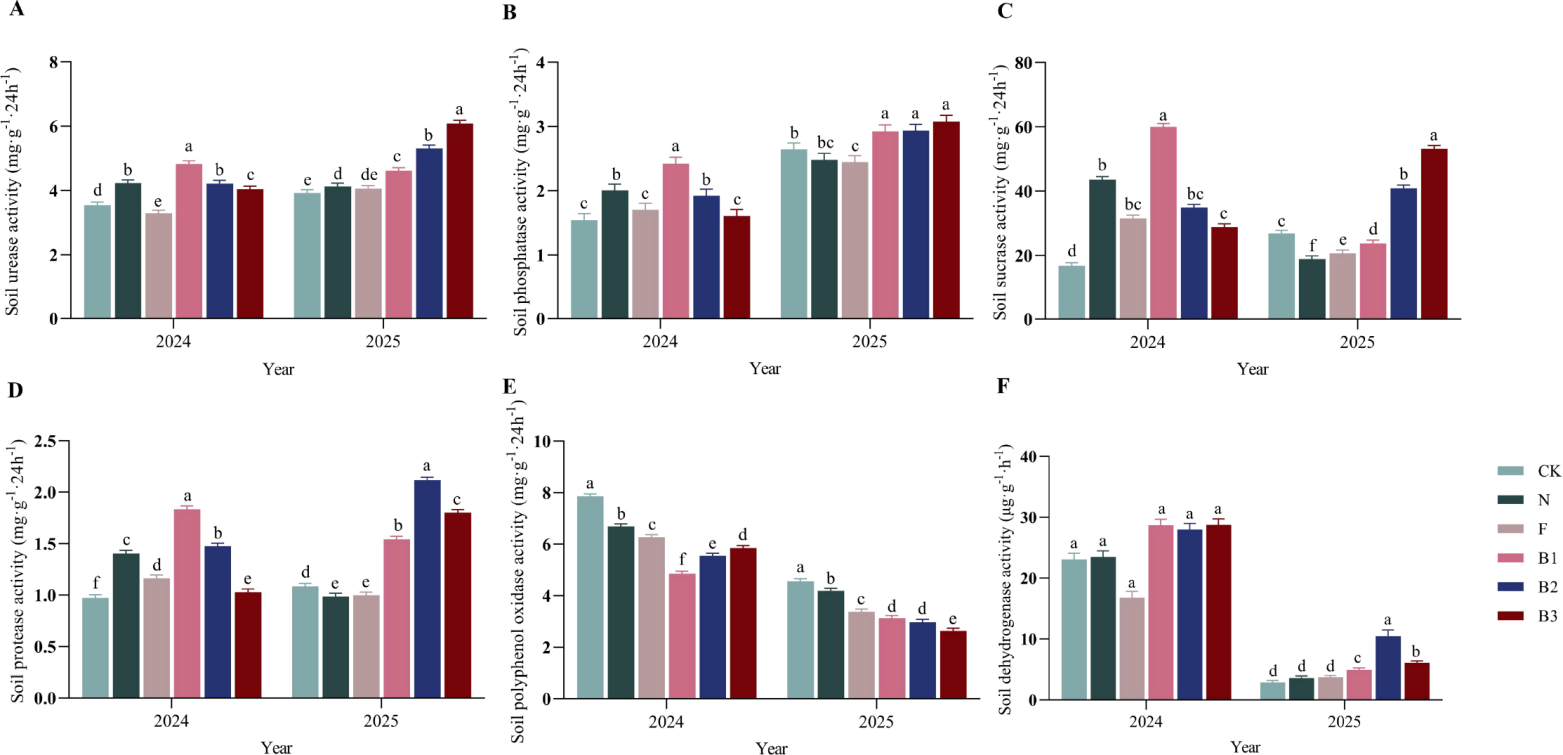
Effect of different fertilization treatments on soil enzyme activities of ‘Saiwaihong’ apple in 2024 and 2025. **(A)** Urease activity. **(B)** Alkaline phosphatase activity. **(C)** Sucrase activity. **(D)** Protease activity. **(E)** Polyphenol oxidase activity. **(F)** Dehydrogenase activity. Data are means ± standard error (n = 3). Different lowercase letters above bars indicate significant differences among treatments within the same year (Tukey’s HSD test, *P* < 0.05). CK: Fertilizer application rate of 0 kg·ha^-1^; N: Manure application rate of 12, 500 kg·ha^-1^; F: Chemical fertilizer application rate of 375 kg·ha^-1^; B1: CB application rate of 10, 750 kg·ha^-1^; B2: CB application rate of 21, 500 kg·ha^-1^; B3: CB application rate of 43, 000 kg·ha^-1^.

Application of the castor bean enzyme biofertilizer (CB) significantly modulated soil enzyme activities, with effects becoming more pronounced in the second year (**Figure 3**). Urease activity responded strongly to CB. In 2024, the B1 treatment exhibited the highest urease activity, exceeding the unfertilized control (CK) by 36.2% (*P <* 0.05). In 2025, urease activity remained relatively stable across treatments, but all CB application rates (B1, B2, B3) sustained significantly elevated levels compared to CK, with increases of 17.5%, 35.4%, and 55.2%, respectively. Phosphatase activity was also enhanced by CB. The B1 treatment resulted in the greatest activity in 2024 (a 56.7% increase over CK). Although overall activity increased in 2025, CB-treated plots consistently outperformed others, with B1, B2, and B3 significantly exceeding CK by 83.5%, 38.0%, and 23.1%, respectively. Sucrase activity peaked under B1 in 2024, with a 258.2% increase over CK, followed by the cattle manure treatment. Despite a general decline in.

sucrase activity across all treatments in 2025, CB applications (B2 and B3) maintained significantly higher levels than other fertilized plots, surpassing CK by 52.3% and 98.0%, respectively. Protease activity showed minor fluctuations in absolute values between years. However, the treatment yielding the highest activity shifted from B1 in 2024 to B2 in 2025, indicating that CB consistently resulted in protease activity levels superior to those of other amendments. Conversely, polyphenol oxidase (PPO) activity was markedly suppressed by CB. Across both years, all CB treatments (B1, B2, B3) showed significantly lower PPO activity than other fertilization regimes, with average reductions of 33.7%, 32.1%, and 33.9% relative to CK, respectively-an effect that was more pronounced in 2025. In contrast to the suppression of PPO, soil dehydrogenase activity (DHA) exhibited a different pattern. While no significant differences were detected among treatments in 2024, the B2 treatment exhibited substantially higher DHA in 2025, exceeding CK by 264.5% (*P <* 0.05). In summary, these results demonstrate that while all fertilization regimes influenced soil enzymatic activity, CB application had the most widespread and pronounced effects. This pattern suggests that CB may possess a greater potential to enhance microbially-mediated nutrient cycling processes compared to conventional fertilizers.

### 3.3 Effects of different fertilization treatments on fruit yields

**Figure 4:**
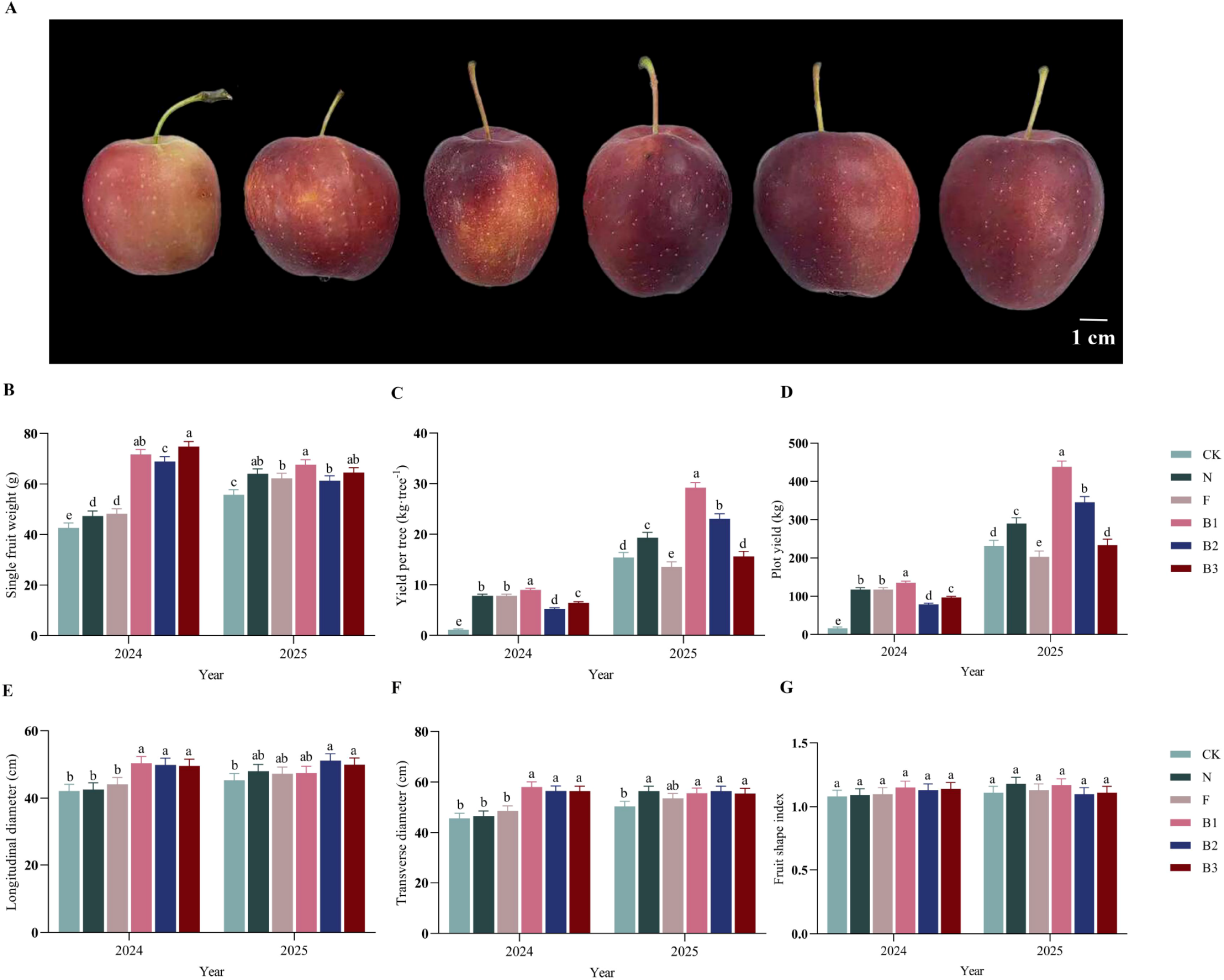
Effect of different fertilization treatments on the yield of ‘Saiwaihong’ apple in 2024 and 2025. **(A)** Fruit weight. **(B)** Yield per tree. **(C)** Plot yield. **(D)** Longitudinal diameter. **(E)** Transverse diameter. **(F)** Fruit shape index. Data are means ± standard error (n = 3). **(G)** Fruit shape index. Different lowercase letters above bars indicate significant differences among treatments within the same year (Tukey’s HSD test, *P* < 0.05). CK, Fertilizer application rate of 0 kg·ha^-1^; N, Manure application rate of 12, 500 kg·ha^-1^; F, Chemical fertilizer application rate of 375 kg·ha^-1^; B1, CB application rate of 10, 750 kg·ha^-1^; B2, CB application rate of 21, 500 kg·ha^-1^; B3, CB application rate of 43, 000 kg·ha^-1^.

Fertilization regimes significantly influenced both the yield components and final yield of ‘Saiwaihong’ apples (**Figures 4A–D**). In 2024, all CB treatments (B1, B2, B3) markedly increased fruit weight, exceeding the unfertilized control (CK) by 68.0%, 61.5%, and 75.4%, respectively. By 2025, fruit weight under all fertilized treatments remained significantly higher than CK, with increases of 14.9% for cattle manure (N), 11.6% for chemical fertilizer (F), and 15.7% to 21.3% for the CB treatments (B1-B3). Yield per tree increased substantially from 2024 to 2025. In 2024, all fertilization treatments resulted in significantly higher per-tree yields than CK, with the B1 treatment showing the highest increase (718.2%). In 2025, the B1 and B2 treatments continued to yield the most, exceeding CK by 89.6% and 49.7%, respectively. Regarding plot-level yield, the beneficial effects of castor bean enzyme biofertilizer were sustained across both years. The B1 and B2 treatments consistently produced significantly higher plot yields than CK, with average annual increases of 403.9% and 213.5%, respectively. These results demonstrate that castor bean enzyme biofertilizer confers a significant and persistent advantage in enhancing the productivity of ‘Saiwaihong’ apple trees.

### 3.4 Effects of different fertilization treatments on fruit quality

#### 3.4.1 Effects of different fertilization treatments on fruit appearance quality

Fertilization regimes significantly affected the size of ‘Saiwaihong’ apples, though the fruit shape index remained largely unchanged (**Figures 4E–G**). In 2024, all CB treatments (B1, B2, B3) markedly increased fruit transverse diameter relative to the unfertilized control (CK), with increases of 19.5%, 18.3%, and 17.6%, respectively. In 2025, transverse diameter was higher under all fertilized treatments than under CK. However, this increase was statistically significant only for the B2 (12.9%) and B3 (10.2%) treatments, with the apparent increases under cattle manure (N, 5.9%), chemical fertilizer (F, 4.4%), and B1 (4.7%) being non-significant. Longitudinal diameter followed a similar pattern. In 2024, the B1, B2, and B3 treatments resulted in significantly greater longitudinal diameter than CK, with increases of 27.1%, 23.6%, and 23.4%, respectively. In 2025, both cattle manure and all CB treatments (B1, B2, B3) maintained significantly higher longitudinal diameter than CK, showing increases of 11.9%, 10.2%, 11.9%, and 10.0%, respectively. Mean longitudinal diameter across all treatments was similar between 2024 and 2025. Despite these significant effects on fruit dimensions, the fruit shape index-a ratio of longitudinal to transverse diameter-exhibited minimal responsiveness to fertilization. In 2024, the B1 treatment yielded the highest index, whereas in 2025, the cattle manure treatment resulted in the highest value. However, none of the fertilization treatments significantly altered the fruit shape index compared to CK in either year, indicating that the proportional increases in fruit dimensions were largely consistent.

#### 3.4.2 Effects of different fertilization treatments on fruit internal quality

**Figure 5:**
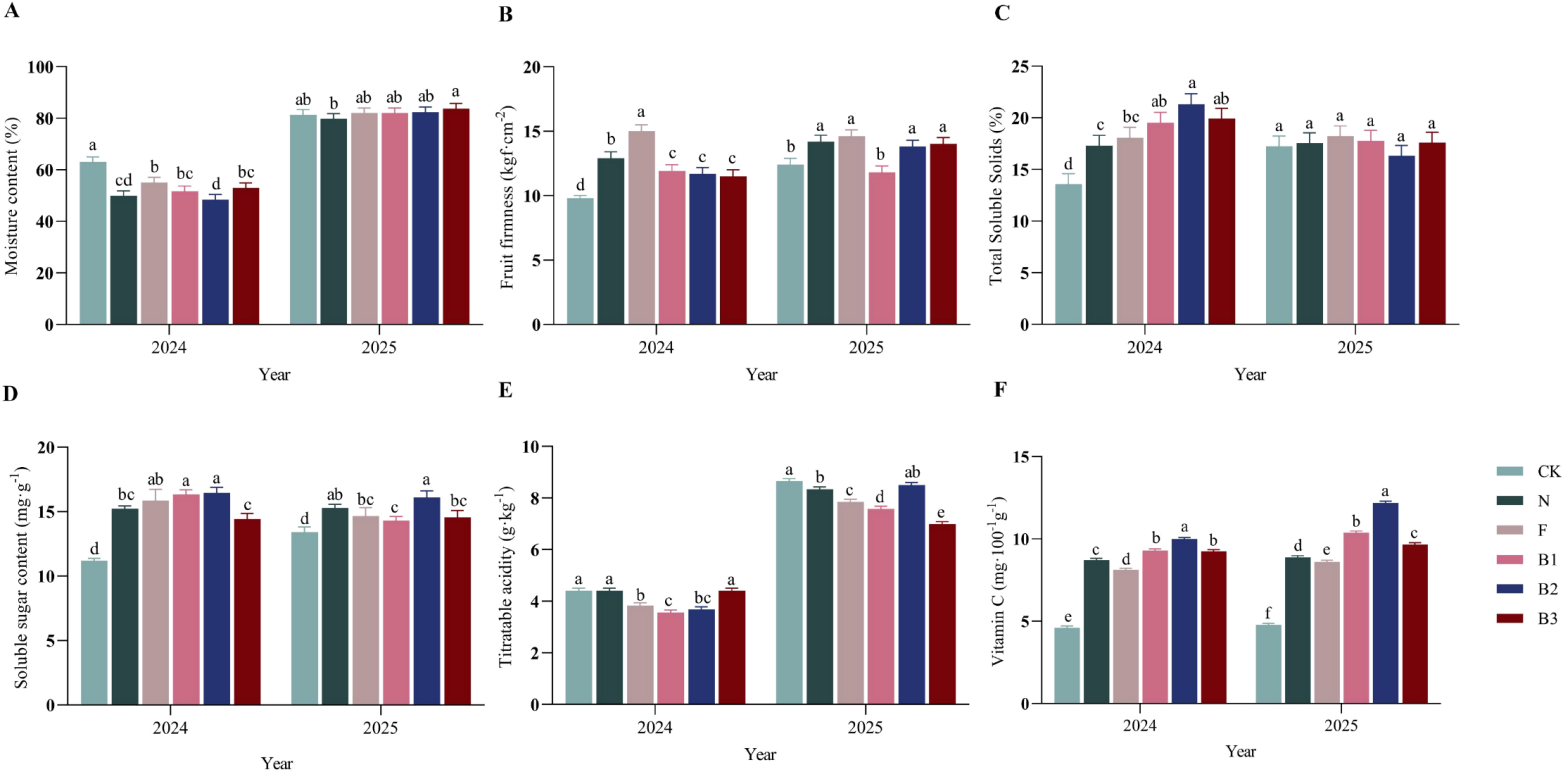
Effect of different fertilization treatments on the quality of ‘Saiwaihong’ apple in 2024 and 2025. **(A)** Fruit moisture content. **(B)** Fruit firmness. **(C)** Soluble solids content. **(D)** Soluble sugar content. **(E)** Titratable acidity content. **(F)** Vitamin C content. Data are means ± standard error (n = 3). Different lowercase letters above bars indicate significant differences among treatments within the same year (Tukey’s HSD test, *P* < 0.05). CK, Fertilizer application rate of 0 kg·ha^-1^; N, Manure application rate of 12, 500 kg·ha^-1^; F, Chemical fertilizer application rate of 375 kg·ha^-1^; B1, CB application rate of 10, 750 kg·ha^-1^; B2, CB application rate of 21, 500 kg·ha^-1^; B3, CB application rate of 43, 000 kg·ha^-1^.

Fertilization treatments differentially influenced the fruit quality parameters of ‘Saiwaihong’ apples, with castor bean enzyme biofertilizer demonstrating superior effects on key nutritional and taste-related traits (**Figure 5**). Fruit moisture content exhibited interannual variation. In 2024, all fertilization treatments resulted in lower moisture content than the unfertilized control (CK). By 2025, moisture content increased substantially across treatments, though no significant differences were detected among fertilization regimes. Fruit firmness was influenced by fertilization regime. In 2024, cattle manure (N) and chemical fertilizer (F) treatments produced the firmest fruits, exceeding CK by 31.6% and 53.1%, respectively, while firmness under CB treatments was comparable to CK. In 2025, N, F, and CB treatments (B2, B3) maintained significantly higher firmness than CK, with increases of 14.5%, 17.1%, 11.3%, and 12.9%, respectively. Firmness values were similar between 2024 and 2025. Soluble solids content (SSC) was markedly enhanced by CB in the first year. In 2024, all CB treatments (B1, B2, B3) resulted in higher SSC than other fertilization regimes, significantly exceeding CK by 43.6%, 56.8%, and 46.5%, respectively. However, SSC values in 2025 were lower than those in 2024 across all treatments, and no significant differences were detected among fertilization regimes in 2025. Soluble sugar content remained relatively stable across the two-year study. In 2024, the B1 and B2 treatments significantly increased soluble sugar content compared to CK, with elevations of 46.1% and 47.0%, respectively. In 2025, the B2 treatment maintained the highest soluble sugar content (16.1 mg·g^-1^), representing a 20.1% increase over CK. Titratable acidity (TA) showed pronounced interannual variation, with substantially higher values in 2025 than in 2024. In 2024, N and B3 treatments exhibited TA levels comparable to CK, while F, B1, and B2 treatments significantly reduced TA by 12.9%, 19.3%, and 16.3% relative to CK, respectively. In 2025, the B3 treatment resulted in the lowest TA, significantly decreasing by 19.2% compared to CK. Vitamin C content was consistently enhanced by CB application across both years. The B1, B2, and B3 treatments increased vitamin C content by average annual margins of 109.4%, 135.9%, and 101.4% over CK, respectively-a substantially greater increase than observed with other fertilizers. Moreover, vitamin C content across all treatments was substantially higher in 2025 than in 2024. In summary, these results demonstrate that castor bean enzyme biofertilizer confers a more comprehensive enhancement of fruit quality in ‘Saiwaihong’ apples than either cattle manure or chemical fertilizer. This was particularly evident in its superior effects on the accumulation of soluble solids, soluble sugars, and especially vitamin C.

### 3.5 Correlation analysis of soil nutrients, soil enzyme activities, and ‘Saiwaihong’ apple yield and quality

**Figure 6:**
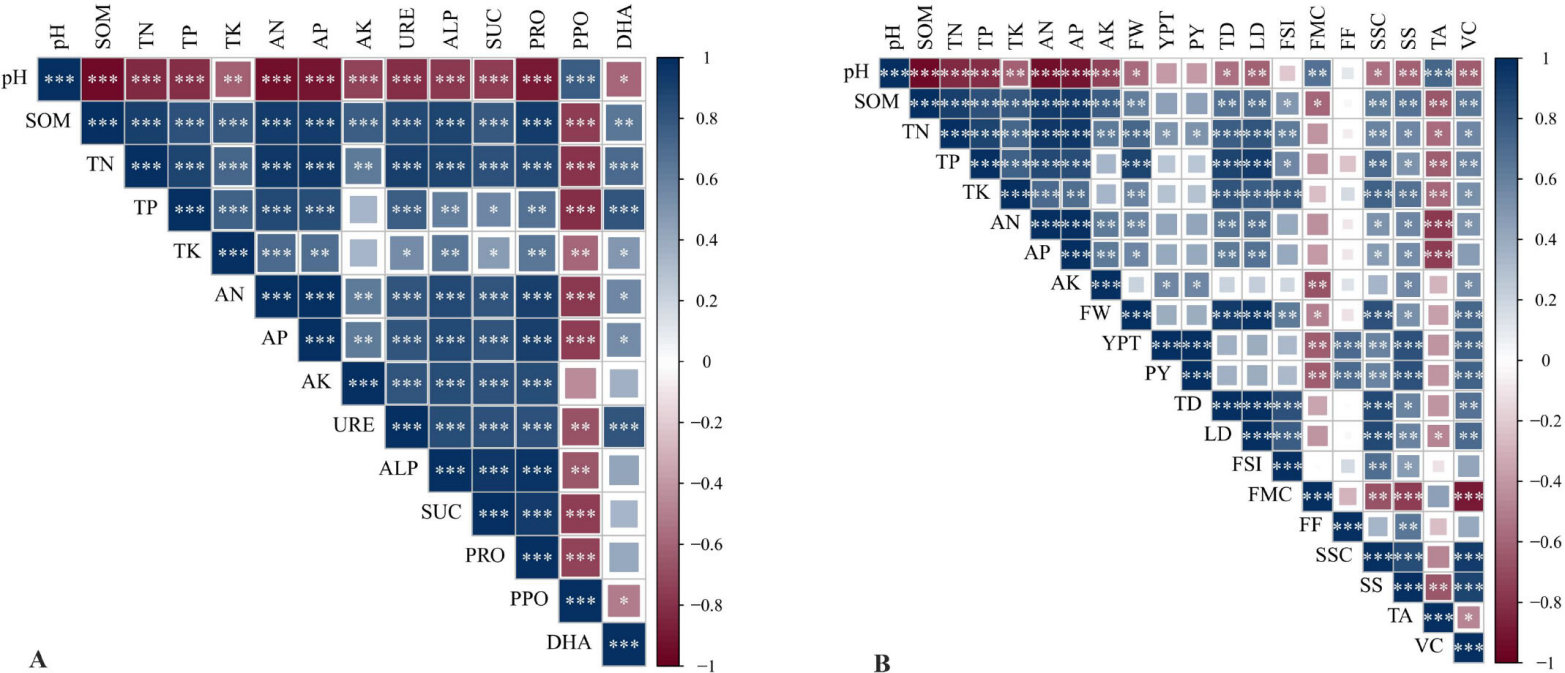
Correlation analysis of soil nutrients and soil enzyme activities with agronomic traits, yield of ‘Saiwaihong’ apple. **(A)** Heat map of correlation analysis between soil nutrients and soil enzyme activity; **(B)** Heat map of correlation analysis between soil nutrients and ‘Saiwaihong’ apple agronomic traits, yield indexes. pH, Soil organic matter (SOM), Total nitrogen (TN), Total phosphorus (TP), Total potassium (TK), Alkaline dissolved nitrogen (AN), Alkaline phosphorus (AP), Available potassium (AK), Urease (URE). Alkaline phosphatase (ALP). Sucrase (SUC). Protease (PRO). Polyphenol oxidase (PPO). Dehydrogenase (DHA). Fruit weight (FW). Yield per tree (YPT). Plot yield (PY). Transverse diameter (TD). Longitudinal diameter (LD). Fruit shape index (PSI). Fruit moisture content (FMC). Fruit firmness (FF). Soluble solids content (SSC). Soluble sugar (SS). Titratable acidity (TA). Vitamin C (VC). The darker the color, the stronger the correlation. Red indicates a negative correlation, while blue indicates a positive correlation. Correlation is significant at the *P* < 0.05, *P* < 0.01 and *P* < 0.001 level.

Correlation analysis revealed distinct patterns among soil properties, enzyme activities, and fruit quality attributes in ‘Saiwaihong’ apple (**Figure 6**). Soil pH emerged as a master regulator, exhibiting highly significant negative correlations with all measured soil nutrients (SOM, TN, TP, TK, AN, AP, AK) and most hydrolase enzymes (URE, ALP, SUC, PRO) (*P <* 0.001), while showing a unique positive correlation with polyphenol oxidase (PPO) activity (*P <* 0.01). A strong positive synergy was observed among soil nutrients and hydrolytic enzymes. Total nitrogen (TN) served as a central hub, positively correlating with TP, TK, available nutrients, and the suite of URE, ALP, SUC, PRO, and DHA (*P <* 0.01). Similarly, available nutrients (AN, AP, AK) were tightly coupled with the activity of URE, ALP, SUC, and PRO (*P <* 0.001), indicating a coordinated nutrient-enzyme system that was consistently suppressed by low pH. Regarding fruit quality, yield parameters (YPT, PY) and physical attributes (FW, TD, LD) showed the strongest and most frequent positive correlations with TN and TP, underscoring the pivotal role of nitrogen and phosphorus availability in determining fruit development. Soluble solids content (SSC), a key quality indicator, was most strongly associated with TK, FW, and fruit dimensions (*P <* 0.001), while titratable acidity (TA) exhibited negative correlations with several soil nutrients, particularly SOM, TP, and TK (*P <* 0.05). Collectively, these correlation patterns demonstrate that the biofertilizer-mediated enhancement of soil nutrient availability and enzymatic activity directly translates into improved yield and quality parameters in ‘Saiwaihong’ apples.

## 4 Discussion

### 4.1 Effects of different fertilization treatments on soil nutrients in ‘Saiwaihong’ apples

Application of castor bean enzyme biofertilizer (CB) fundamentally reshaped the soil nutrient landscape in the ‘Saiwaihong’ apple orchard, with effects that diverged markedly from conventional fertilizers and intensified over the two-year experimental period. These findings align with growing recognition that organic-based amendments exert profound effects on soil biogeochemistry, particularly in nutrient-poor sandy substrates characteristic of the Horqin sandy land (Shu et al., 2022; Chiodi et al., 2025). Similarly, Jakhro and Li (2025) demonstrated that organic fertilizer application significantly enhances soil nitrogen, phosphorus, and carbon levels in apple orchards across China and Pakistan, further supporting the notion that organic amendments consistently improve soil nutrient status across diverse orchard systems. In this study, the most striking effect of CB application was the consistent pH reduction across both years (**Figure 2A**). While the initial pH decline in 2024 may partially reflect the acidic nature of CB itself, the sustained reduction in 2025 suggests a fundamental shift in soil acid-base chemistry. This persistent acidification likely results from enhanced nitrification of ammoniacal nitrogen released from CB, a well-documented process wherein proton release during NH_4_^+^ oxidation progressively lowers pH (Wang et al., 2025). This interpretation is supported by concomitant elevation in alkaline-hydrolyzable nitrogen (AN) under CB treatments, particularly B2 in 2025, indicating active nitrogen transformation (**Figure 2F**). Given the strongly alkaline initial soil conditions, this pH moderation toward a more neutral range potentially enhanced bioavailability of phosphorus and micronutrients often limiting in calcareous soils (Bohórquez-Sandoval et al., 2025; Zhang et al., 2025). Concurrent with pH modulation, CB dramatically elevated soil organic matter (SOM), with B1 and B2 increasing SOM by an average of 70.6% and 50.9% over CK across both years (**Figure 2B**). This enhancement substantially exceeds that reported for conventional organic amendments in apple orchards (Rathinapriya et al., 2025; Jakhro and Li, 2025). Wang et al. (2025a) observed soil organic carbon increases of 4–9 g·kg^-1^ with chicken manure-based composites, whereas our CB treatments achieved SOM elevations equivalent to approximately 10–15 g·kg^-1^ organic carbon. The superior performance likely stems from CB’s dual nature as both microbial inoculant and organic substrate. The beneficial microorganism consortium (*Bacillus subtilis*, *Saccharomyces cerevisiae*, *Spirulina platensis*) may accelerate decomposition of castor meal’s complex organic polymers while contributing microbial necromass-a key precursor for stable soil organic carbon formation (Li M et al., 2024). The slight SOM decline from 2024 to 2025 across all treatments reflects typical mineralization dynamics following organic amendment incorporation; yet CB-treated plots maintained significantly higher SOM than controls, indicating sustained organic matter accrual. Nutrient-specific responses to CB reveal distinct mechanistic pathways. The pronounced and persistent elevation of total nitrogen (TN) under all CB treatments (averaging 51.0-89.6% above CK) likely reflects both direct nutrient input and enhanced biological nitrogen fixation (Zheng et al., 2024a; Li M et al., 2025) (**Figure 2C**). The seven microbial strains in CB inoculant, particularly *Spirulina platensis*, may contribute to asymptotic N_2_ fixation, though this hypothesis requires confirmation through nitrogenase activity assays. The even more dramatic increases in available phosphorus (AP)-exceeding CK by 2407.6% and 3009.9% under B1 and B2, respectively-cannot be explained by direct P input alone (**Figure 2G**). This remarkable AP elevation likely results from multiple synergistic mechanisms: (1) organic anion competition for phosphate adsorption sites on soil colloids (Kerner et al., 2023). (2) pH-mediated desorption of occluded P (Ahmed et al., 2024b). (3) enhanced phosphatase activity mineralizing organic P pools (Li et al., 2020). Sustained AP elevation into 2025 suggests CB induced a lasting shift in phosphorus cycling, rather than merely supplying soluble P that would be rapidly fixed in calcareous soils. Temporal dynamics of available potassium (AK) provide insight into differential nutrient release patterns among amendments. In 2024, cattle manure (N) exhibited the highest AK, reflecting immediate K supply. However, by 2025, B2 surpassed all others, increasing AK by 405.7% over CK (**Figure 2H**). This delayed K availability peak under CB may reflect gradual K release from the castor meal matrix as microbial decomposition progresses, coupled with reduced K fixation due to modified soil pH and organic matter coating of K-fixing clay minerals (Xu et al., 2025; Li et al., 2017). Similar patterns occur in organic-based composites where sustained nutrient release aligns better with tree phenological demands than pulse supply from conventional fertilizers (Priya et al., 2024). Notably, total potassium (TK) remained unaffected by any fertilization treatment throughout the study, underscoring the distinction between total and available nutrient pools (**Figure 2E**). TK primarily reflects soil parent material and is largely insensitive to short-term fertilization, whereas available pools reflect dynamic equilibrium between solubilization, fixation, and plant uptake (Li J et al., 2024). The decoupling between TK (invariant) and AK (highly responsive) reinforces that agronomic significance resides in available nutrient fractions, which CB treatments demonstrably enhanced.

The superior performance of moderate CB rates (B1: 10, 750 kg·ha^-1^; B2: 21, 500 kg·ha^-1^) relative to the highest rate (B3: 43, 000 kg·ha^-1^) warrants consideration. While B3 supplied the greatest total nutrient inputs, it did not consistently yield the highest available nutrient concentrations or fruit quality parameters. This pattern suggests threshold effects in organic amendment application, beyond which additional inputs may not translate to proportional increases in plant-available nutrients-a phenomenon observed where excessive organic matter inputs can temporarily immobilize nutrients or disrupt soil biological equilibria (Ling et al., 2025; Omokaro et al., 2024). Moderate CB rates may achieve optimal stoichiometric balance between carbon and nutrient inputs, facilitating rather than overwhelming microbial mineralization processes (Li M et al., 2025). Collectively, these results demonstrate that castor bean enzyme biofertilizer mediates soil nutrient enrichment through mechanisms distinct from either conventional compound fertilizer or unprocessed cattle manure. The combination of organic substrate, microbial inoculum, and associated enzymes creates synergistic effects on nutrient availability extending beyond simple nutrient addition. This aligns with broader literature indicating that properly formulated organic amendments enhance nutrient cycling efficiency in orchard systems, potentially reducing dependence on synthetic fertilizers while maintaining or improving soil fertility (Xie et al., 2025a; Huang et al., 2025). For the sandy, low-organic-matter soils of the Horqin region, such amendments represent a particularly valuable strategy for building long-term soil health and supporting sustainable apple production.

### 4.2 Effects of different fertilization treatments on soil enzymatic activity in ‘Saiwaihong’ apples

Castor bean enzyme biofertilizer (CB) profoundly modulated the soil enzymatic environment, with effects that diverged qualitatively from conventional amendments and intensified over the two-year study. These findings indicate that CB functions beyond a mere nutrient source, acting as a catalyst that restructures soil biochemical potential and enhances nutrient cycling capacity (Hu et al., 2024; Zhou et al., 2023). The most striking observation was the sustained elevation of hydrolytic enzyme activities-urease, phosphatase, sucrase, and protease-under CB treatments. Urease activity, which catalyzes urea hydrolysis to ammonia and CO_2_, responded robustly to CB application, with B1, B2, and B3 treatments maintaining significantly elevated levels across both years (**Figure 3A**). This enhancement cannot be attributed solely to direct enzyme content of the CB inoculant, which contained eight exogenous enzymes including urease. Rather, the sustained elevation-particularly the continued increase in B3 from 2024 to 2025-suggests a systemic shift in indigenous microbial community metabolic capacity, consistent with priming effects documented in enzyme-amended soils (Liu et al., 2020). Concomitant elevation in alkaline phosphatase activity (56.7-83.5% above CK) further supports this interpretation, as phosphatase synthesis is typically induced under organic phosphorus availability-conditions created by CB application (**Figure 3B**). The magnitude of phosphatase enhancement substantially exceeds that reported for conventional organic amendments (Islam et al., 2026). Agbede (2025) observed 20-35% increases with chicken manure, whereas our CB treatments achieved nearly double that effect. Sucrase activity exhibited the most dramatic response to CB, with B1 increasing activity by 258.2% over CK in 2024-an effect size unprecedented in orchard soil amendment literature (**Figure 3C**). Sucrase mediates sucrose hydrolysis to glucose and fructose, representing a critical bottleneck in soil carbon cycling and plant-available carbohydrate supply (Li Q et al., 2024). This extraordinary stimulation likely reflects CB’s dual nature as substrate and inoculum: castor meal provides abundant polysaccharides that induce microbial production of saccharolytic enzymes, while introduced microorganisms (*Bacillus subtilis*, *Saccharomyces cerevisiae*) directly contribute to the extracellular enzyme pool (Li M et al., 2025). The subsequent decline in sucrase activity across all treatments in 2025, though CB-treated plots maintained significantly higher levels than controls (52.3-98.0% above CK), suggests an initial pulse of microbial activity followed by stabilization at an elevated equilibrium-a pattern characteristic of successful soil biological regeneration. The contrasting behavior of polyphenol oxidase (PPO) provides additional mechanistic insight. PPO catalyzes phenolic compound oxidation to quinones, a process associated with lignin degradation and humification (Andrés et al., 2023). Across both years, all CB treatments exhibited significantly suppressed PPO activity relative to other fertilization regimes, with suppression becoming more pronounced in 2025 (**Figure 3E**). While initially counterintuitive, this suppression is mechanistically consistent with reduced phenolic substrate availability resulting from CB-mediated pH modification. CB reduced soil pH from strongly alkaline to near-neutral conditions. Phenolic compounds exhibit pH-dependent solubility and bioavailability, with alkaline conditions favoring solubilization and thus substrate availability for PPO (Pasquet et al., 2024). By moderating soil pH toward neutrality, CB may have reduced phenolic solubility, diminishing substrate-induced PPO synthesis (Wang et al., 2025b). Alternatively, suppression may reflect shifts in fungal community composition, as PPO is primarily produced by *white-rot fungi* and *Actinobacteria* differentially sensitive to pH (Jiang et al., 2021). Regardless of mechanism, the functional consequence is clear: CB shifted the soil enzymatic balance from oxidative, lignin-degrading processes toward hydrolytic, nutrient-mineralizing processes-a shift characteristic of high-fertility agricultural soils (Liao et al., 2022). Dehydrogenase activity (DHA), an intracellular enzyme indicator of overall microbial metabolic activity, exhibited a distinct temporal pattern. While no treatment differences were detectable in 2024, B2 showed dramatically elevated DHA in 2025 (264.5% above CK), with B1 and B3 showing intermediate, non-significant increases (**Figure 3F**). This delayed response likely reflects time required for microbial community restructuring following amendment addition. This interpretation aligns with nutrient availability patterns, wherein B2 consistently outperformed B3 despite receiving half the total nutrient input, underscoring that “more is not necessarily better” in organic amendment management. The intermediate CB rate appears to achieve optimal stoichiometric balance between carbon and nutrient inputs, maximizing microbial metabolic efficiency and hydrolytic enzyme synthesis without inducing negative feedbacks associated with organic matter excess (Wang et al., 2024). Strong positive correlations between available nutrients (AN, AP, AK) and hydrolytic enzyme activities (URE, ALP, SUC, PRO) provide direct evidence for functional coupling between enzyme-mediated nutrient mineralization and plant-available nutrient pools (**Figure 6A**). This coupling was particularly tight for phosphatase activity and available phosphorus, consistent with phosphatases’ established role in organic P mineralization (Li et al., 2021b). The extraordinary AP elevations under CB (2407.6-3009.9% above CK) cannot be explained by direct P input alone; rather, they reflect synergistic interaction between enhanced phosphatase activity and pH-mediated desorption of mineral-bound P. This dual mechanism-biological and physicochemical-explains why CB achieved AP levels far exceeding conventional fertilizer despite providing comparable total P inputs. Similarly, strong correlation between urease activity and AN indicates that CB-mediated urease enhancement directly contributed to nitrogen availability. Correlation patterns also illuminated relationships between soil enzymatic activity and fruit quality (**Figure 6A**). Strongest associations between enzyme activities and fruit quality parameters were observed for vitamin C content, which correlated positively with urease and phosphatase activities. This finding suggests that nutritional quality enhancement conferred by CB is mediated, at least partially, through improved soil biological functioning rather than direct nutrient supply alone (Weiss and Gruda, 2025). In contrast, while chemical fertilizer (F) rapidly supplied crop-available nutrients, it failed to sustain soil enzyme activity (e.g., urease and ALP activities under F reverted to CK levels by 2025). Cattle manure (N), on the other hand, only modestly enhanced soil organic matter (SOM) without appreciably affecting soil enzyme activity. This divergence indicates that the nutrient composition and pH-modulating effects of the CB biofertilizer create a positive feedback loop: lowered pH alleviates micronutrient limitations, while elevated hydrolase activities (particularly urease and ALP) accelerate N and P turnover, ultimately enhancing ‘Saiwaihong’ apple quality.

### 4.3 Effects of different fertilization treatments on the yield and quality of ‘Saiwaihong’ apples

The differential responses of yield and fruit quality to fertilization regimes provide compelling evidence that castor bean enzyme biofertilizer (CB) confers advantages beyond those achievable with conventional fertilizers or unprocessed organic amendments. These findings align with research demonstrating that bio-organic fertilizers can simultaneously enhance productivity and nutritional quality in fruit crops (Xia et al., 2025; Zheng et al., 2024b).

### 4.3.1 Effects of different fertilization treatments on the yield of ‘Saiwaihong’ apples

The dramatic yield increases under CB treatments-particularly the 403.9% and 213.5% average annual increases in plot yield under B1 and B2, respectively-substantially exceed those typically reported for organic amendments in apple production systems (Li Q et al., 2024). The magnitude of yield response likely reflects compounded benefits of CB’s dual action: immediate nutrient supply combined with sustained improvements in soil biological functioning that enhance nutrient availability over time (Rani et al., 2025). The temporal pattern of yield response-with B1 highest in 2024 but B2 superior in 2025-provides insight into CB-mediated yield dynamics (**Figure 4D**). The initial advantage of B1 likely reflects rapid mineralization of readily available nutrients, consistent with immediate increases in available nitrogen and phosphorus in 2024. However, sustained superiority of B2 in 2025 suggests that moderate CB rates achieve a more favorable balance between nutrient supply and microbial immobilization, avoiding temporary nitrogen sequestration that can occur with excessive organic matter inputs (Xie et al., 2025b). This interpretation is supported by soil enzyme data, wherein B2 exhibited highest dehydrogenase activity in 2025, indicating elevated microbial metabolic activity without carbon overflow effects hypothesized for B3. Concurrent enhancement of single fruit weight under all CB treatments (15.7-21.3% above CK in 2025) indicates yield gains were achieved through increased individual fruit size rather than merely fruit number-a critical distinction for market valuation (**Figures 4A, B**). This pattern contrasts with some organic fertilization studies wherein yield increases were primarily driven by fruit number with stable or reduced individual fruit mass. Maintenance of fruit size under CB likely reflects balanced nutrient supply through gradual organic matter mineralization, supporting sustained fruit expansion without nutrient dilution effects associated with high-yielding conventional systems (Zhao et al., 2021; Bu et al., 2025).

#### 4.3.2 Effects of different fertilization treatments on the quality of ‘Saiwaihong’ apples

The superior and sustained enhancement of vitamin C content under CB treatments (average annual increases of 101.4-135.9% above CK) represents the most striking quality outcome of this study (**Figure 5F**). This magnitude of vitamin C elevation substantially exceeds that reported for foliar calcium applications in ‘Snick’ apples and for boron plus humic acid treatments in ‘Anna’ apples (Xu et al., 2025; Wang et al., 2025). The consistency of this effect across both years and all CB rates suggests a fundamental shift in fruit primary metabolism rather than a transient response to specific nutrient inputs. Two mechanistic pathways may explain this phenomenon. First, the improved soil biological functioning under CB may enhance root uptake of micronutrients (e.g., copper, zinc, manganese) that serve as cofactors for ascorbate biosynthesis enzymes (Elrys et al., 2024). Second, the moderate reduction in soil pH toward neutrality likely increased the bioavailability of these micronutrients, which are often limiting in calcareous soils (Mitsuta et al., 2025). The strong positive correlations between vitamin C content and both urease and phosphatase activities support the interpretation that enhanced soil biological activity translates into improved fruit nutritional quality. Notably, despite supplying higher nitrogen levels, the F treatment failed to increase vitamin C content, likely due to its inability to persistently enhance soil enzyme activity (**Figure 3**). The N treatment, although it increased soil organic matter, neither lowered soil pH nor introduced functional microbes, leading to only a marginal rise in vitamin C content. The soluble solids content (SSC) response exhibited a more complex pattern, with dramatic CB-mediated increases in 2024 that were not sustained in 2025 (**Figure 5C**). This temporal variability may reflect the known sensitivity of sugar accumulation to environmental conditions, particularly water availability during fruit maturation (Ren et al., 2023). The 2024 growing season, with its favorable precipitation distribution, may have allowed fuller expression of CB’s effects on carbohydrate metabolism. Alternatively, the elevated SSC in 2024 may partially reflect a concentration effect from reduced fruit moisture content observed that year, whereas the concurrent increases in both fruit size and SSC in 2025 indicate genuine enhancement of sugar accumulation capacity. The sustained elevation of soluble sugars under B2 in 2025 supports this interpretation and aligns with findings that organic amendments can enhance sugar metabolism through improved potassium nutrition (Shu et al., 2022) (**Figure 5D**). The reduction in titratable acidity (TA) under CB and chemical fertilizer treatments, particularly pronounced in 2025 for B3 (**Figure 5E**), reflects the well-established inverse relationship between nitrogen availability and organic acid accumulation in fruit (**Figure 6B**). Enhanced nitrogen supply promotes conversion of organic acids to sugars during ripening, improving the sugar:acid ratio that is critical for sensory quality (Huang et al., 2021). This pattern is consistent with observations in multiple fruit crops wherein balanced fertilization reduces excessive acidity while maintaining or enhancing sugar content (Mehta et al., 2025).

These findings contribute to the emerging understanding that bio-organic fertilizers can simultaneously enhance soil health, crop productivity, and nutritional quality-addressing the triple bottom line of sustainable agriculture. The results align with meta-analyses indicating that organic amendments typically increase soil organic matter and microbial activity while maintaining yields comparable to conventional systems, though the magnitude of yield enhancement observed here exceeds typical values. The specific benefits of castor bean-based formulations may relate to the unique composition of castor meal, including its high protein content, diverse mineral elements, and bioactive compounds that stimulate microbial activity. Several questions remain for future investigation. First, the mechanisms linking enhanced soil biological activity to fruit quality improvements require elucidation through targeted metabolomic and transcriptomic analyses. Second, the optimal CB application rate may vary with soil type, climate, and orchard age; multi-location trials are needed to develop site-specific recommendations. Third, the economic analysis of CB adoption-considering both input costs and potential premium pricing for enhanced fruit quality-will be essential for grower decision-making. Finally, long-term studies are needed to assess whether the benefits of CB accumulate over multiple years, potentially reducing the need for supplemental fertilization and further enhancing the sustainability of ‘Saiwaihong’ apple production systems.

## 5 Conclusion

This study demonstrates that castor bean enzyme biofertilizer (CB) at the moderate application rate (21, 500 kg·ha^-1^; B2) optimally enhances soil properties, enzymatic activity, and crop performance in ‘Saiwaihong’ apple production. CB application reduced soil pH from strongly alkaline to near-neutral conditions, elevated soil organic matter by 50.9-70.6%, and dramatically increased available phosphorus and nitrogen availability. These improvements were accompanied by sustained elevation of hydrolytic enzyme activities (urease, phosphatase, sucrase, protease) and suppression of polyphenol oxidase, indicating a qualitative shift toward nutrient-mineralizing soil processes. The B2 treatment achieved superior yield enhancement through increased individual fruit size, while concurrently elevating fruit vitamin C content by 101.4-135.9%-an effect size substantially exceeding those reported for conventional amendments. Strong correlations between soil enzyme activities and fruit quality parameters indicate that enhanced soil biological functioning directly translates into improved nutritional quality. To achieve high-yield and high-quality cultivation of ‘Saiwaihong’ apples while reducing reliance on synthetic inputs, we recommend CB application at 21, 500 kg·ha^-1^. This bio-organic fertilizer represents a promising strategy for building long-term soil health and supporting sustainable orchard management in the sandy, low-organic-matter soils of the Horqin region, providing theoretical guidance for green agriculture development.

## Data Availability

The datasets presented in this study can be found in online repositories. The names of the repository/repositories and accession number(s) can be found in the article/**Supplementary Material**.
